# The effect of hydro-alcoholic extract of *Ceratonia Silique L.* on spermatogenesis index in rats treated with cyclophosphamide: An experimental study

**DOI:** 10.18502/ijrm.v13i4.6892

**Published:** 2020-04-30

**Authors:** Firouzeh Sadeghzadeh, Azizeh Sadeghzadeh, Saeed Changizi-Ashtiyani, Sepideh Bakhshi, Farideh Jalali Mashayekhi, Mehry Mashayekhi, Hossein Poorcheraghi, Ali Zarei, Mostafa Jafari

**Affiliations:** ^1^Traditional and Complementary Medicine Research Center, Arak University of Medical Sciences, Arak, Iran.; ^2^Department of Endocrinology and Female Infertility, Reproductive Biomedicine Research Center, Royan Institute for Reproductive Biomedicine, ACECR, Tehran, Iran.; ^3^Student Research Committee, Arak University of Medical Sciences, Arak, Iran.; ^4^Department of Physiology, Shiraz University of Medical Sciences, Shiraz, Iran.; ^5^Department of Neurology, University of Minnesota, Minneapolis, USA.

**Keywords:** Cyclophosphamide, Ceratonia siliqua, Spermatogenesis, Rat, Spermatogenesis indexes.

## Abstract

**Background:**

The aim of this study was to investigate the antioxidant effects of *Ceratonia* extract on improving the toxicity induced by cyclophosphamide (CP) on spermatogenesis. **Materials and Methods:** 54 male Wistar rats (4 months old) weighing 200-250 gr were randomly divided into 6 groups (n = 9/each):

**Objective:**

“group 1 (control) underwent the normal diet and water; group 2 (sham) received 2 ml/day normal saline; group 3 (positive control) received 300 mg/kg/day *Ceratonia* extract; group 4 *(Ceratonia + CP)* received *Ceratonia* extract (300 mg/kg/day) + 5 mg/kg/day CP (Endoxan, baxter oncology gmbh, Germany) after 4 hr; group 5 (CP) received 5 mg/kg/day CP + normal saline 4 hr after it; and group 6 (CP + *Ceratonia*) received *Ceratonia* extract (300 mg/kg/day) 4 hr after 5 mg/kg/day CP." 24 hr after the last gavage, heart blood sampling was performed to measure the levels of malondialdehyde (MDA), ferric reducing antioxidant power, testosterone, luteinizing hormone, and follicle-stimulating hormone. The left caudal epididymis was cut in the Ham's F10 and the released spermatozoa were used to analyze sperm parameters. The histology of the right testes was studied using stereological techniques and the left testes were used to measure the level of tissue MDA and ferric reducing antioxidant power.

**Results:**

A significant increase in the mean level of MDA (p = 0.013) was seen in the CP compared to the control group. Sperm motility (p = 0.001) and count (p = 0.002), serum and tissue total antioxidant (p ≤ 0.001) and serum testosterone levels (p = 0.019) decreased in the CP compared to the control group. *Ceratonia* extract could significantly prevent the adverse effects of CP on sperm motility (p < 0.001), the mean levels of tissue MDA (p = 0.018), serum total antioxidant (p = 0.045), and testosterone (p < 0.001).

**Conclusion:**

The *Ceratonia* extract can modify the reproductive toxicity of CP in rat due to the presence of antioxidant compounds.

## 1. Introduction

Spermatogenesis is a complex physiological process that involve a fine balance between germ cell proliferation, differentiation, and apoptosis in the testes but several factors such as drug treatment, toxins, and environmental toxins can have negative effects on it (1). Cyclophosphamide (CP) is an immunosuppressive agent which is used in organ transplants as well as for the treatment of the nephrotic syndrome. Despite its many clinical applications, it has side effects. Many studies have confirmed the incidence of oligospermia and azoospermia, as well as the elimination of spermatogenic cycles in adult males treated with this drug. Long-term use of this drug causes changes in the levels of reactive thiobarbituric acid (TBA) and antioxidant levels of cells/tissues, which are indicators of reactive oxygen species (ROS) production (2-4). Previous studies have shown that CP decreases testosterone levels, testosterone activity, and aromatase biosynthesis, which is probably due to oxidative stress damage and alteration of the gene expression pattern in Leydig cells (4, 5). Although the exact mechanism by which CP causes testicular injury is still unknown, numerous studies have shown that the production of free radicals disrupts the balance of antioxidants, and pro-oxidants are responsible for the CP toxicity in the testis and sperm (4, 6, 7). The CP metabolite, acrolein, increase lipid peroxidation and oxidative damage, and Sertoli cells apoptosis. Furthermore, sperm cells are more sensitive to lipid peroxidation due to the abundance of polyunsaturated fatty acids residing in their plasma membrane and a lack of antioxidant content in their cytoplasm (6). Oxidative stress in sperm induced immobility, reduced fertilization capacity, significantly increase frequencies of sperm cell DNA damage and sperm apoptosis, which have a significant negative impact on male fertility (8).

On the other hand, traditionally, herbal treatment regimens have been used to treat infertility (4). *Ceratonia silique* *(Ceratonia)* is a medicinal herb that grows widely in the world and in some regions of Iran. The structure of this plant consists of 40% simple carbohydrates, 1% fat, and about 3-4% of proteins. In addition, it contains polyphenols, especially tannins, fiber, minerals such as potassium, sodium, calcium, iron, phosphorus, and vitamins such as vitamins E, D, B6, niacin, folic acid, selenium, antioxidant properties that reduces the oxidative stress and is also effective in preventing and treating chronic inflammation due to the presence of polyphenols as a complementary therapeutic supplement (9-11). Previous reports have shown that *Ceratonia* decreases the levels of malondialdehyde (MDA) while it increases the level of enzymes superoxide dismutase, and catalase (11, 12). Also, Vafaei and colleagues showed that *Ceratonia* increases the mean value of germinal epithelium thickness as well as thiol levels (13).

Considering that in some areas it has been shown that carob has a positive effect on male fertility and spermatogenesis, the purpose of this study was to investigate the efficacy and protective effect of hydroalcoholic extract of *Ceratonia* on sperm parameters (motility, morphology, viability, count) and spermatogenesis indexes (ubule Differentiation Index (TDI), Spermiogenesis Index (SPI), Meiosis Index (MI), and Sertoli cell Index (SCI), and the mean levels of malondialdehyde (MDA), ferric reducing antioxidant power, luteinizing hormone, and follicle-stimulating hormone (FSH) in rats exposed to CP.

## 2. Materials and Methods

### Animals and studied groups

This experimental study was performed on 54 male Wistar rats (4 months old) weighing 200-250 gr. Animals were purchased from the Laboratory Animal Breeding Center of Baqiyatallah University of Medical Sciences (Iran) and were randomly divided into six groups (n = 9): group 1 (control) underwent the normal diet and water; group 2 (sham) received 2 ml/day normal saline; group 3 (positive control) received 300 mg/kg/day *Ceratonia* extract (14); group 4 *(Ceratonia + CP)* received *Ceratonia* extract (300 mg/kg/day) + 5 mg/kg/day CP (Endoxan, baxter oncology gmbh, Germany) after 4 hr; group 5 (CP) received 5 mg/kg/day CP (15, 16) + normal saline 4 hr after it; and group 6 (CP + *Ceratonia*) received Ceratonia extract (300 mg/kg/day) 4 hr after 5 mg/kg/day CP. CP and *Ceratonia *extract were administered for 28 days by gavage. Treatment period was selected on the basis of previous studies that demonstrated 5 mg/kg/day of CP treatment for 28 days induces toxicity in the testis tissue (15, 16).

### Preparation of medicinal herb extract

The plant fruits used in the present study were collected from Kazeroon (Fars, Iran) and after assuring the genus and species and storing some of it in plant's research institute herbium was used to prepare the extract. To prepare the hydro-alcoholic extract, the crumbs were broken, then the seeds were separated and crushed to obtain a soft powder. One kilogram of the resulting powder 50/50 with 96% ethanol alcohol was water-soaked for 72 hr. The solution was then placed in the oven at 40°C until the water and alcohol evaporated and brown concentrated extract remained. To obtain different concentrations, the desired amounts of the extract were dissolved in distilled water (17).

### Surgical procedure

The rats were anesthetized by ether 24 hr after the last gavage. The left caudal epididymis were isolated to examine the sperm parameters and the right caudal epididymis were isolated to examine the mass. Blood samples were also collected from the animal's heart to assess the MDA levels, ferric reducing antioxidant power, testosterone, LH, and FSH. The testicles were carefully isolated and weighed. For histological studies, the right testes were fixed in a fixative solution of modified Davidson's fluid for 1 wk and the left testes were stored at 80°C to evaluate the tissue level of MDA and FRAP (1).

### Removal of epididymis

Immediately after anesthesia, the left caudal epididymis was isolated and placed in a plate containing 10 ml of Ham's F10 culture medium. Then, epididymis was cut by the sterile blade and the plate was placed in a 37°C incubator for 5 min. The released sperms were used to calculate sperm motility and morphology (18).

### Sperm analysis 

#### Sperm motility 

For evaluation of sperm motility, 10 μl of the sperm suspension was transferred to Neobar slide and examined under light microscope (Nikon Labophot 2) with a magnification of 200×. Each sperm was classified either as progressive motion, motion, or motionless (static), and also the percentage was calculated (1).

#### Sperm count

The sperm suspensions were prepared by diluting the culture medium containing sperm with 2% formaldehyde fixative. To evaluate the number of sperm, Neobar lam was used. After putting 10 μl of sperm suspension on Neobar slide, sperms with head, mid area, and tail were counted by light microscope and 40× magnification. After counting the sperm in squares, their number in 1 μl sample size was calculated (19).

#### Sperm morphology

To study the morphology of sperms, the sperm suspension were stained with eosin/nicrosine and a thin expansion of the sample was made on a slide. For each sample, 100 sperm with 100× magnification light microscope were examined and the percentage of normality was expressed (19).

#### Stereological study

The orientator method was used to obtain isotropic uniform random (IUR) sections. After tissue processing and embedding, 20 µ sections were prepared and stained by Hematoxylin and eosin stain method (1).

#### Spermatogenesis indexes

In total, 100 round seminiferous tubule cross sections in each testis tissue were selected randomly and all cells were counted. TDI, SPI, MI, and SCI as spermatogenesis indexs were investigated.

TDI is the percentage of isolating tubes containing at least three spermatogenesis differentiated from Type B spermatogonial cells. SPI is the percentage of tubules that obtain the overall rate of spermatogenesis. MI is the ratio of the number of spermatid cells to spermatocyte. SCI is the ratio of the total number of spermatogonial cells and spermatocyte to the number of Sertoli cells (1).

### Biochemical evaluations

#### Evaluation of testes MDA level

The left testes tissue was hemogenised in KCL and was mixed with a stock solution (2 ml, 12% w/v trichloroacetic acid 0.375% w/v TBA, and 0.25 mol /l hydrochloric acid) and then the tissue MDA was calculated by measuring TBARS (TBA-reactive substances) and the absorbance was measured at 532 nm. Results were expressed as micromole per grams of tissue samples (1).

#### Measurement of serum MDA level 

One ml. of serum was mixed with 2 ml of stock solution (12% w/v trichloroacetic acid 0.375% w/v TBA, and 0.25 mol /l hydrochloric acid), and the new solution was placed in boiling Ben-Marie for 15 min. After removing and cooling, microtube was centrifuged for 10 min. The precipitate was removed and its absorbance was determined at 532 nm against reagent blank. The concentration of MDA was calculated using its extinction coefficient, which is 1/56 * 10${}^{5}$ M${}^{-1}$ cm${}^{-1}$ and expressed in terms of nmol per milliliter (nmol/ml) (1).

#### Serum testosterone determination 

Blood testosterone concentrations were measured according to the ELISA kit (Monobind Testosterone ELISA kit, USA) and in ng/mol. 10 μl of the sample with 50 μl conjugate solution was poured into the well and then the testosterone solution and the anti-testosterone solution were added and incubated for 60 min at room temperature. The wells were then washed with deionized water and 100 μl of substrate were added to each well and incubated for 15 min at room temperature and in a dark environment. Then, by the addition of 50 μl of stop solution, the reaction ended and its absorption was read with an ELISA device at 450 nm.

#### Total antioxidant capacity method by FRAP method

In the FRAP measurement method, 100 μl of homogeneous plasma or homogeneous tissue was poured into the cuvettes, then 3 ml of the FRAP ready to add to the cuvettes was added and after 4 min their absorption at 593 nm against Blanc was read. Then, using the regression formula obtained in the standard curve, the FRAP values of blood samples were obtained in micromoles per gram (20).

#### Measurement of serum LH and FSH level

The serum LH and FSH levels were evaluated with LH and FSH ELISA kits (Rat Luteinizing Hormone, LH ELISA Kit ZB-10179-R9648, FSH, FSH ELISA Kit ZB-10182-R9648 Zellbio Inc., Germany) according to the manufacturer's instructions.

### Ethical consideration

All animals were kept and studied under the same conditions in terms of access to water and food, light (12 hr), and temperature (21-23°C). The study protocol was approved by ethics committee of Arak University of Medical Sciences, Arak, Iran (Code: IR.ARAKMU.REC.1396.208).

### Statistical analysis 

Data were analyzed using the Statistical Package for Social Sciences (SPSS), version 16/0 (SPSS Inc., Chicago, Illinois, USA) and one-way ANOVA and Tukey statistical tests. Mean ± SD difference was considered significant at p < 0.05 level.

## 3. Results

In this study, we analyzed the changes in body weight, sperm parameters, stress oxidative, and hormones of the hypothalamic*-*pituitary*-*gonadal axis, stereological studies, and biochemical data.

### Study of spermatogenesis indexes and sperm count

The spermatogenesis indexes showed no significant difference in all different groups. The number of sperm in CP-treated rats was significantly reduced compared to the control group. While the Ceratonia resulted in an increase in the number of sperm in the CP + Ceratonia group and the Ceratonia + CP group, this increase was higher in the CP + Ceratonia group than in the Ceratonia + CP group (Table I).

### Motility and sperm morphology

In rats treated with CP, the mean percentage of progressive sperms was significantly lower than the control group, while the mean percentage of immotile sperm and non-progressive sperms showed a significant increase. The *Ceratonia* was able to increase the average percentage of progressive sperms and decrease the average percentage of non-progressive and immotile sperm. No significant difference was observed in the sperm morphology in all experimental groups (Table II).

### Biochemical data

#### Evaluation of MDA and total antioxidant capacity

A significant increase was observed in testis MDA level in CP-treated rats compared to the control group, while *Ceratonia* reduced its level in CP + *Ceratonia* and CP + *Ceratonia* group compared to the CP group in which CP + *Ceratonia* decreased further. The mean serum total antioxidant capacity level in CP-treated rats was significantly lower than the control group.

On the other hand, mean levels of total antioxidant capacity of serum and tissue in rats treated with CP were significantly lower than the control group. *Ceratonia* significantly increased the total serum antioxidant level in *Ceratonia* group and a significant increase was found in the total tissue antioxidant level in *Ceratonia*, CP + *Ceratonia*, and the *Ceratonia* + CP groups when compared to the CP group (Table III).

#### Testosterone levels, LH, and FSH serum levels

Serum testosterone level reduced significantly in CP group compared to the control group). Whereas it showed an increase in the CP + Ceratonia and Ceratonia + CP group when compared to the CP group, but this increase in Ceratonia + CP group was not significant. Serum levels of LH and FSH showed no significant difference in all different groups (Table IV).

#### Body, testicular, and epididymis weight

There was a significant difference in the mean body weight between different groups after the end of the treatment. The average of body weight after treatment with CP decreased significantly compered to Ceratonia group. Body weight in the CP group showed a significant decrease compared to the control group (Table V).

**Table 1:** Average Sertoli cell coefficient (SCI) and sperm count in different groups


**Groups**	**Sertoli cell coefficient (SCI)**	**Sperm count (×10${}^{6}$/mL)**
**Control**	14.15 ± 1.6	30 ± 3.4†
**Sham**	13.74 ± 1.9 (p = 0.99)	28.4 ± 3.4 (p = 0.82)†
**CP**	11.2 ± 0.6 (p = 0.02)	17.4 ± 1.5 (p = 0.002)*
*Ceratonia*	11.86 ± 1.7 (p = 0.09)	25.2 ± 3.5 (p = 0.01)
*Ceratonia* + CP	11.17 ± 1.7 (p = 0.03)*	20.8 ± 1.9 (p < 0.001)*
**CP + ** ***Ceratonia*** ****	11.64 ± 1.3 (p = 0.05)	23 ± 1.6 (p = 0.002)*†
Data presented as Mean ± SD (One-way ANOVA and Tukey's test, p < 0.05) *Significant difference between the specified group and the control group; †Significant difference between the specified group and CP group		

**Table 2:** Comparison of the mean sperm motility and sperm count in different groups


**Groups**	**Progressive motility (%)**	**Non-progressive motility (%)**	**Immotile (%)**
**Control**	78.2 ± 3.8†	15 ± 3.5†	6.8 ± 2.2†
**Sham**	80.4 ± 5.7 (p = 0.77)†	13.4 ± 4 (p = 0.85)†	7.8 ± 2.7 (p = 0.98)†
**CP**	39.2 ± 1.3 (p <0.001)*	35.6 ± 1.1 (p < 0.001)*	25.2 ± 1.3 (p < 0.011)*
*Ceratonia*	79.2 ± 4.4 (p = 0.99)†	16.6 ± 3.6 (p = 0.98)†	6.2 ± 2.9 (p = 0.98)†
*Ceratonia* + CP	70.2 ± 7.1 (p = 0.006)†	16.8 ± 4.2 (p = 0.97)†	13 ± 4.1 (p < 0.001)*†
*CP + Ceratonia*	70.4 ± 4.2 (p = 0.008)†	14.4 ± 4.2 (p = 0.99)†	15.4 ± 2.5 (p < 0.001)*†
Data presented as Mean ± SD (One-way ANOVA and Tukey's test, p < 0.05) *Significant difference between the specified group and the control group; †Significant difference between the specified group and CP group			

**Table 3:** Comparison of mean MDA concentration and total antioxidant capacity in testicular and serum tissues in different groups


**Groups**	**MDA tissue concentration (nmol/mg)**	**Serum MDA concentration (nmol/ml)**	**Total tissue antioxidant capacity level (nmol/mg)**	**Total serum antioxidant capacity level (nmol/ml)**
**Control**	0.08 ± 0.06†	0.45 ± 0.1	0.57 ± 0.08†	0.62 ± 0.19†
**Sham**	0.09 ± 0.01 (p = 0.99)†	0.57 ± 0.02 (p = 0.30)	0.58 ± 0.05 (p = 1.00)†	0.62 ± 0.09 (p = 1.00)†
**CP**	0.13 ± 0.02 (p = 0.013)*	0.62 ± 0.07 (p = 0.07)	0.46 ± 0.05 (p ≤ 0.001)*	0.54 ± 0.11 (p ≤ 0.001)*
*Ceratonia*	0.04 ± 0.02 (p = 0.51)†	0.50 ± 0.29 (p = 0.55)	0.55 ± 0.03 (p = 0.56)	0.65 ± 0.1 (p = 0.005)†
*Ceratonia* + CP	0.09 ± 0.02 (p = 0.99)†	0.51 ± 0.24 (p = 0.55)	0.49 ± 0.09 (p = 0.05)	0.60 ± 0.21 (p = 0.04)†
**CP** *** +*** **** ***Ceratonia*** ****	0.05 ± 0.005 (p = 0.80)†	0.40 ± 0.17 (p = 0.93)	0.50 ± 0.04 (p = 0.02)	0.59 ± 0.15 (p ≤ 0.001)†
Data presented as Mean ± SD (One-way ANOVA and Tukey's test, p < 0.05) *Significant difference between the specified group and the control group; †Significant difference between the specified group and CP group				

**Table 4:** Average concentration of testosterone, LH, and FSH in different groups


**Groups**	**Serum testosterone concentration (ng/ml)**	**Mean serum LH concentration (IU/L)**	**Mean serum FSH concentration (mIU/L)**
**Control**	1.47 ± 0.1†	2.1 ± 0.8	2.24 ± 0.9
**Sham**	1.66 ± 0.04 (p = 0.74)†	2.05 ± 0.5 (p = 0.95)	2.19 ± 0.1 (p = 0.96)
**CP**	0.51 ± 0.4 (p < 0.01)*	1.46 ± 0.1 (p = 0.06)	1.47 ± 0.3 (p = 0.05)
*Ceratonia*	1.59 ± 0.4 (p = 0.99)†	1.89 ± 0.2 (p = 0.29)	1.56 ± 0.6 (p = 0.09)
*Ceratonia* + CP	0.69 ± 0.5 (p = 0.06)*	1.74 ± 0.5 (p = 0.05)	1.82 ± 0.9 (p = 0.12)
*CP + Ceratonia*	1.22 ± 0.5 (p = 0.07)	2.05 ± 0.5 (p = 0.98)	2.22 ± 1.4 (p = 0.98)
Data presented as Mean + SD (One-way ANOVA and Tukey's test, p < 0.05) *Significant difference between the specified group and the control group; †Significant difference between the specified group and CP group			

**Table 5:** Comparison of the mean body weight, testis, and epididymis of mice in different groups


**Groups**	**Average initial weight of mice (gr)**	**Average weight of mice at the end of treatment (gr)**	**Average testicular weight of mice (gr)**	**Average weight of rats epididymis (gr)**
**Control**	207 ± 4.8	285 ± 22.9†	1.49 ± 0.11	0.58 ± 0.03
**Sham**	204 ± 5.4 (p = 1.00)	280 ± 17.6 (p = 0.99)	1.48 ± 0.09 (p = 1.00)	0.56 ± 0.04 (p = 0.99)
**CP**	204 ± 7.7 (p = 1.00)	250 ± 18.7 (p < 0.04)*	1.52 ± 0.13 (p = 0.78)	0.57 ± 0.03 (p = 1.00)
*Ceratonia*	213 ± 9.7 (p = 0.77)	301 ± 17 (p = 0.08)*†	1.6 ± 0.13 (p = 0.04)	0.61 ± 0.04 (p = 0.99)
*Ceratonia* + CP	211 ± 14 (p = 1.00)	281 ± 11.4 (p = 0.99)	1.51 ± 0.13 (p = 0.92)	0.56 ± 0.05 (p = 0.99)
*CP + Ceratonia*	203 ± 11.2 (p = 1.00)	266 ± 13.5 (p = 0.13)	1.57 ± 0.16 (p = 0.29)	0.56 ± 0.05 (p = 0.91)
Data presented as Mean + SD (One-way ANOVA and Tukey's test, p < 0.05) *Significant difference between the specified group and the control group; †Significant difference between the specified group and CP group				

## 4. Discussion

In this study, we demonstrated that *Ceratonia *in the CP + *Ceratonia*-treated group could increase spermatogenesis indexes, sperm count, and motility, and serum testosterone level and decrease MDA.

As our data showed, treatment with CP in adult rats leads to decrease of serum testosterone level which is aligned with other studies (4, 16). Ghosh and colleagues demonstrated that CP causes a significant decrease in the transcription of genes encoding enzymes responsible for the biosynthesis of testosterone, and decreases aromatase, serum testosterone level, and germ cells spermatogenesis. These changes may be due to the oxidative damage and a change in the CP -induced gene expression pattern in Leydig cells. By reduction of the activity of these enzymes, the testosterone level will decrease (16). It seems that the increase in testosterone level in the *Ceratonia*-treated group is due to its direct effect on Leydig cells and on testosterone biosynthesis. The results of this study showed an increased level of MDA and decreased serum total antioxidant capacity in CP-treated rats, which is in accordance with other studies (21-23). *Ceratonia* reduced the level of MDA and increased the total antioxidant capacity of serum (5, 16, 24).

In Shalizar Jalali and co-worker's study, a reduction in stereological parameters, spermatogenesis activity, sperm count, serum antioxidant capacity and testosterone level (25), in Torabi and colleagues' study a reduction in sperm count and motility (26), and in Anan and co-worker's study an apoptosis in the germ cells (15) as a result of treatment with CP was reported. As Hamzeh and colleagues demonstrated, CP causes accumulation of unconsumed lipids in the spermatogenic cells and therefore destroys spermatogenic cells (4). On the other hand, it causes an increase in pro-apoptotic genes such as the P53, Bax, and Cytochrome C and a reduction in anti-apoptotic Bcl2 gene transcripts (27).

CP reduces the level of SOD, CAT, glutathione, glucose-6-phosphate dehydrogenase, glutathi-one peroxidase, and vitamins E and C, which lead to an increase in the sensitivity of cells to oxidative damage, and increases the lipid peroxidation. It also increases its final product; MAD, by decreasing the level of nuclear factor erythroid 2-related factor 2 (Nrf2), a vital regulatory gene for proteins that cause cell survival under oxidative stress, increases the level of ROS in these cells. Nrf2 defects in cells increase the susceptibility to various oxidants (5).

Elhalim and colleagues showed that *Ceratonia *extract exhibits antioxidant properties and reduces electrophoretic changes in CAT and glutathione peroxidase patterns, which may be due to the presence of polyphenols (such as gallic acid, epigalacethicin 3-galate, and apicachtin-3-galate), flavanols, parantoicinidins, and tannins (28). This eliminates the free radicals, and thus reduces the peroxidation response, subsequently reduce MDA levels (24).

CP affects the activity of antioxidant enzymes by altering the structure of the protein. The CP and its active metabolite, acrolein, oxidize the thiol group with antioxidant enzymes and reduces the enzyme activity. The production of ROS and the formation of lipid peroxidation, as a result of oxidative stress, is a reason to reduce serum total capacity in CP-treated animals, which was similar in the present study (28).

In a study by Mahdiani and colleagues, *Ceratonia* powder was used in 500 mg capsules in 60 men with asthenospermia. In this study, the hydroalcoholic extract of this plant was used at a dose of 300 mg/kg BW, as well in the duration of the intervention. The results showed that the average number and concentration of sperms and the percentage of total sperm motility increased but no significant changes were observed in testosterone levels (29).

As our data showed, treatment with CP in adult rats leads to decrease in sperm motility which is in accordance with other studies (6, 22, 29). Sperm motility is mainly depended on the sperm tail lenght and intracellular levels of adenosine triphosphate (ATP) in the mid-piece (1); therefore, this decrease in sperm motility may be due to the disorder of sperm flagellum function or disturbances in ATP regeneration in sperm as a result of ROS production (6). It was seen that *Ceratonia* treatment increased significantly the sperm motility. So, due to the reduction of ROS, it may be related to antioxidant property of *Ceratonia* that is involved in the protection of sperm cells in CP-treated animals.

In the present study, the mean serum testosterone concentration in the CP group was reduced, while this level was not altered in the CP + *Ceratonia* group, which could be due to the effect of *Ceratonia* in preventing the reduction of testosterone level as a side effect of CP when used.

Arachidonic acid in the *Ceratonia* plant and its metabolites can increase the production of annular adenosine monophosphate and thereby stimulate testosterone production. On the other hand, arachidonic acid plays a major role in the testicular steroidogenesis (30). The other compound contained in *Ceratonia* is an aspartic acid that can increase the production of testosterone as a second peak in Leydig cells by increasing the synthesis of annular adenosine monophosphate (31). Also, *Ceratonia* contains trace elements such as Iron, manganese, copper and zinc which are co-factors of antioxidant enzymes (24) that boost antioxidant system in CP exposure.

Our data revealed a significant decrease in body weight following the treatment with CP compared to the control group. Other studies have also indicated that treatment with CP decreases body weight (22, 32, 33). CP probably affected the amount of fat due to low appetite, preventing protein synthesis. The reduction of protein and fat mass may have caused weight loss. However, Razak and colleagues demonstrated no changes in body weight in the CP-treated group compared to the control group (6). The inconsistent results of the effects of CP on body weight in the previous studies are possibly due to different dose, route of administration and duration of treatment and also, to the sensitivity of the animals used. The weight gain as a result of treatment with *Ceratonia* can be attributed to the antioxidant properties of this plant. Another test observation was the strengthening of testosterone production as a result of the effect of the plant's polyphenolic compounds on Leydig cells (34).

## 5. Conclusion

The findings of this study showed that CP imposes severe tissue and cell damage on testicular tissue and sperm motility and reduces spermatogenesis. On the other hand, hydroalcoholic extract of the *Ceratonia* plant, due to high amounts of vitamin E, various polyphenols and selenium, which is an antioxidant, can prevent the stress-oxidative damage caused by CP. Therefore, its application in therapeutic regimens including CP is suggested.

##  Conflict of Interest

The authors declare that they have no conflict of interest.
